# Studies on Preparation of Poly(3,4-Dihydroxyphenylalanine)-Polylactide Copolymers and the Effect of the Structure of the Copolymers on Their Properties

**DOI:** 10.3390/polym8030092

**Published:** 2016-03-18

**Authors:** Dongjian Shi, Jiali Shen, Zenghui Zhao, Chang Shi, Mingqing Chen

**Affiliations:** The Key Laboratory of Food Colloids and Biotechnology Ministry of Education, School of Chemical and Material Engineering, Jiangnan University, Wuxi 214122, China; djshi@jiangnan.edu.cn (D.S.); jnshenjiali@126.com (J.S.); zhaozenghui419@163.com (Z.Z.); stonebest@icloud.com (C.S.)

**Keywords:** bio-based copolymer, polylactide, 3,4-dihydroxyphenylalanine, degradability, biocompatibility

## Abstract

Properties of copolymers are generally influenced by the structure of the monomers and polymers. For the purpose of understanding the effect of polymer structure on the properties, two kinds of copolymers, poly(3,4-dihydroxyphenylalanine)-*g*-polylactide and poly(3,4-dihydroxyphenylalanine)-*b*-polylactide (PDOPA-*g*-PLA and PDOPA-*b*-PLA) were designed and prepared by ring-opening polymerization of lactide with pre-prepared PDOPA as the initiator and the amidation of the functional PLA and PDOPA oligomer, respectively. The molecular weight and composition of the copolymers could be adjusted by changing the molar ratio of LA and DOPA and were confirmed by gel permeation chromatography (GPC) and proton nuclear magnetic resonance (^1^H NMR) spectra. The obtained copolymers with graft and block structures showed high solubility even in common organic solvents. The effects of the graft and block structures on the thermal and degradation properties were also detected. The PDOPA-*g*-PLA copolymers showed higher thermal stability than the PDOPA-*b*-PLA copolymers, due to the PDOPA-*g*-PLA copolymers with regular structure and strong π-π stacking interactions among the intermolecular and intramolecular chains. In addition, the degradation results showed that the PDOPA-*g*-PLA copolymers and the copolymers with higher DOPA composition had quicker degradation speeds. Interestingly, both two kinds of copolymers, after degradation, became undissolved in the organic solvents because of the oxidation and crosslinking formation of the catechol groups in the DOPA units during degradation in alkaline solution. Moreover, fluorescent microscopy results showed good biocompatibility of the PDOPA-*g*-PLA and PDOPA-*b*-PLA copolymers. The PDOPA and PLA copolymers have the potential applications to the biomedical and industrial fields.

## 1. Introduction

Being aware of the environmental pollution and resource shortage, many investigations on the synthesis and modification of bio-based and natural polymers have been reported over the past decades [1,2,3,4,5]. Bio-based polymers are degradable and have potential applications as adhesives, absorbents, cosmetics, drug delivery carriers, and food packaging products in various fields [6,7,8,9,10]. Poly(lactic acid) (or polylactide, PLA), an aliphatic polyester, that is derived from 100% renewable resources, have attracted significant attention because they have excellent biocompatibility and suitable physicochemical properties [11,12,13,14,15]. However, there is still a great requirement to improve other properties, such as thermal and mechanical properties for increasing the scopes of biomedical or environmental plastic fields. Copolymerization of PLA with other monomers or polymers has been reported to be a useful way for improving the thermal and mechanical properties [16,17]. There are a few studies to prepare the copolymers based on LA and the co-monomer (polymer) [18,19,20,21]. In these examples, introducing rigid monomers (polymers), such as 3,4-dihydroxycinnamic acid (DHCA), their thermal stability and mechanical strength properties would be improved [22,23], whereas, the monomer with a long alkyl chain that was copolymerized with LA could enhance their ductility [24]. Therefore, the properties of the PLA-based copolymers were influenced by the structure of the co-monomers and polymers. Generally, the types of the copolymers, such as the graft and block copolymers, might also affect the polymer properties, except the influence factor of the co-monomer’s structure. However, there are few reports to investigate and compare the properties of the PLA-based graft and block copolymers containing the same co-monomer by changing the segments in the backbone and side chains.

3,4-Dihydroxyphenylalanine (DOPA), a specialized amino acid, is commonly found in many marine organisms and can be used to the treatment of neutral disorders such as Parkinson’s disease [25,26]. DOPA has unique characteristics, such as easy oxidation and adhesion on metals, ceramics, organics, and polymers [27,28,29,30,31]. In an effort to duplicate these characteristics of DOPA in synthetic polymers, DOPA was incorporated into synthetic polymers on side chains or end groups to make them with well adhesive and self-healing properties [32,33]. The formed DOPA polymers will provide important insights into their safety and degradability for the applications as imaging agents, surface treatments and coatings [34,35]. Thus, by combination of the advanced properties of PLA and DOPA, their copolymer might have high performances on thermal, degradable, and other properties. Since DOPA has the hydroxyl, carboxyl, and amine groups, it is very easy to be introduced into the PLA chains by different methods to form the graft and block copolymers. In our previous reports, we have prepared hyperbranched PDOPA polyester with good degradation and biocompatibility by polycondensation [36]. In addition, block-like PDOPA-*b*-PLA copolymer was also synthesized through amidation reaction with the pre-prepared functional PLA and PDOPA [37]. The PDOPA-*b*-PLA copolymer showed well adhesive property to coat on the silica nanorods for efficient drug loading and release. However, the effect of the copolymer structures on the polymer properties is still unknown.

**Scheme 1:**
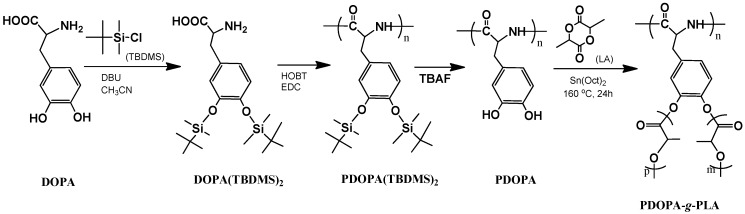
Synthesis of the PDOPA-*g*-PLA copolymer.

**Scheme 2:**
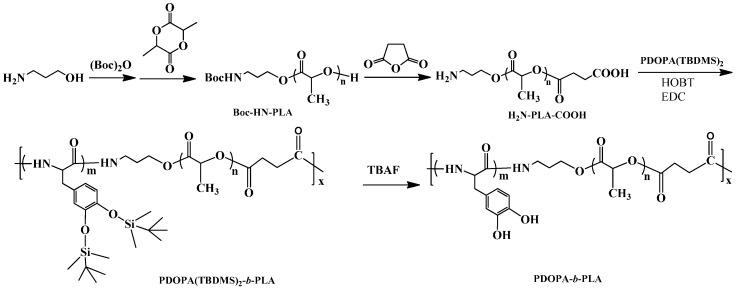
Synthesis of the PDOPA-*b*-PLA copolymer.

To achieve the objective, two type copolymers, PDOPA-*g*-PLA and PDOPA-*b*-PLA with precise structures were designed and synthesized. The PDOPA-*g*-PLA copolymer with the comb-like structure was prepared by ring-opening polymerization (ROP) of lactide via pre-prepared PDOPA polyamide as initiator (Scheme 1). Moreover, the PDOPA-*b*-PLA copolymer with the linear structure was synthesized by amidation of PDOPA and multi-functional PLA (Scheme 2). The effects of the copolymer structures on the solubility, thermal property, degradability, and biocompatibility of the copolymers were investigated in detail.

## 2. Experimental Section

### 2.1. Materials

Lactide (Tokyo Chemical Industrial Co., LTD., Tokyo, Japan) was recrystallized from ethyl acetate, and then dried *in vacuo* at room temperature for 24 h. DOPA (99%), Boc-*t*-butoxycarbonyl (99%), *tert*-butyldimethylsilyl chloride (TBDMS, 97%), 1,8-diazabicyclo[5.4.0]undec-7-ene (DBU, 99%), tetrabutyl ammoniumfluoride (TBAF, 99%), 1-(3-dimethylaminopropyl)-3-ethylcarbodiimide hydrochloride (EDC, 99%), and hydroxybenzotriazole (HOBT, 99%) were obtained from Aladdin Reagent Co., Ltd. (Shanghai, China) and used without purification. Stannous octanoate (Sn(Oct)_2_) was purchased from Sinopharm Chemical Reagent Co., Ltd. (Shanghai, China), and used as received. Methanol, succinic anhydride, ethanol, chloroform, dichloromethane (DCM), and amino propanol were bought from Sinopharm Chemical Industrial Co. Ltd. (Shanghai, China). Dulbecco’s modified eagle medium (DMEM), fetal bovine serum (FBS), and penicillin-streptomycin (P/S) were purchased from Gibco (Thermo Fisher, Shanghai, China). NIH/3T3 cells were purchased from Biomedical Co., Ltd., Suzhou, China. Fluorescein diacetate (FDA) was purchased from Amersco (Beijing, China).

### 2.2. Synthesis of PDOPA(TBDMS)_2_

Three grams of DOPA was added into a stirred solution of TBDMS (6.28 g) in anhydrous acetonitrile. The colorless suspension was cooled in an ice bath for 10 min and addition of DBU (6.5 mL) was subsequently dropped into the above solution [37,38]. After further reaction in the ice bath for 4 h, it was stirred at room temperature for an additional 20 h. The colorless DOPA (TBDMS)_2_ was obtained by evaporating the solvent *in vacuo* (Scheme 1), washed with chloroform, and further purified with methanol/acetonitrile by three dissolving/precipitation cycles.

Then, 2.12 g of DOPA(TBDMS)_2_ was added into 50 mL DCM containing 1.91 g of EDC, 2.73 g of HOBT and 10 mL TEA, and the mixture was kept at room temperature for 24 h. PDOPA(TBDMS)_2_ of a white color was obtained by extraction with DCM three times and dried *in vacuo*.

### 2.3. Synthesis of PDOPA-g-PLA Copolymer

PDOPA(TBDMS)_2_ (2 g) was dissolved in 10 mL THF and 1 mL TBAF and maintained the reaction for 2–4 h for deprotection of the hydroxyl groups in DOPA (Scheme 1). The PDOPA polyamide was obtained after washing with distilled water three times and dried at room temperature.

Then, a proportion of LA was added into the toluene solution containing the initiator PDOPA and Sn(Oct)_2_ (3 wt %). The mixture was kept at 130 °C for 1 h, and subsequently heated to 160 °C for 4 h. The PDOPA-*g*-PLA copolymer was dissolved in CHCl_3_ and precipitated into diethyl ether for three cycles.

### 2.4. Synthesis of PDOPA-b-PLA Copolymer

Functional H_2_N-PLA-COOH polymer was synthesized as per the previous reports [37,39]. H_2_N-PLA-COOH (1.5 g) and a certain amount of PDOPA(TBDMS)_2_ was dissolved in methanol, and reacted at 25 °C for 24 h in the presence of EDC and HOBT as catalysts. The PDOPA-*b*-PLA copolymer was obtained by using TBAF to remove the TBDMS groups (Scheme 2), and was then further purified by dissolving in acetone to remove the unreacted PLA and dialysis in ethanol to remove PDOPA.

### 2.5. Hydrolytic Degradation of the Copolymers

The degradation of the as-synthesized PDOPA-*g*-PLA and PDOPA-*b*-PLA copolymers was studied by an acceleration test in phosphate buffers of pH 10.2 at 37 °C. The copolymer films (approximately 20 mm × 10 mm × 1 mm in size) that were fabricated with the solvent evaporation method in CHCl_3_ were suspended in excess of the buffer solution. The buffer solution was replaced at some point to maintain the pH value at a constant. The degradation behavior was determined by the weight change of the samples during hydrolytic degradation. All experiments were repeated in triplicate.

### 2.6. Cell Adhesion of the Copolymers

The PDOPA-*g*-PLA and PDOPA-*b*-PLA copolymer films were placed into a plastic dish with 3.5 cm diameter (5 × 10^4^ cells/well) in complete DMEM (with 10% fetal bovine serum and penicillin-streptomycin supplemented) suspension in a 5% CO_2_ incubator at 37 °C for 24 h. Finally, the NIH/3T3 cells were stained with FDA, which could cleave to a green fluorescent product by metabolically-active cells. The density of the cells that adhered on each scaffold was measured from randomly selected views of each film, which was observed at 100-fold magnification by a fluorescent microscopy (Nikon 80i, Japan).

### 2.7. Characterization

Fourier transform infrared spectroscopy (Nicolet iS50 FTIR, Thermo Fisher Scientific, Madison, WI, USA) was performed by casting samples in dichloromethane onto NaCl plates. Proton nuclear magnetic resonance (^1^H NMR) analysis was recorded on a BRUKER 400 AVANCEIII spectrometer (Bruker, Fällanden, Switzerland) with deuterated chloroform (CDCl_3_) as the solvent, and an internal tetramethylsilane (TMS) as reference standard. Number-average molecular weight (*M*_n_), weight-average molecular weight (*M*_w_), and polydispersity index (PDI) were measured by gel permeation chromatography (GPC, Waters 1515, Waters, Milford, MA, USA) using mono-dispersed polystyrene as standards and DMF ad eluent. The copolymers were eluted with tetrahydrofuran (THF) through a linear Styragel HR2 column (Waters, 7.8 mm × 300 mm) at a flow rate of 1 mL/min. Differential scanning calorimetry (DSC) was performed under nitrogen using a heat/cool/heat cycle at a heating rate of 10 °C/min from 25 to 300 °C, on a PE Instruments DSC8000 (PE, Cincinnati, OH, USA) with aluminum pan. Thermogravimetric analysis (TGA) was performed with a TGA 1100SF apparatus from Mettler Toledo International Trading Co., Ltd. (Mettler Toledo, Greifensee, Switzerland) Instruments under nitrogen flow at a heating rate of 10 °C/min from 25 to 600 °C. The decomposition temperature (*T*_d,5%_) value was calculated from the temperature at 5% weight loss of decomposition. The contact angle of the copolymer sheet was measured using an OCA 15EC (Dataphysics Co., Ltd., Filderstadt, Germany) with ultra-pure water droplets. The average value was obtained by placed several drops on the sheet surface. The surface morphology of the film before and after degradation was observed by an S-4800 scanning electron microscope (SEM, Hitachi Limited, Tokyo, Japan) operating at 15 kV. All the samples were sputter-coated with gold to minimize sample charging.

## 3. Results and Discussion

### 3.1. Structural Characterization

**Figure 1:**
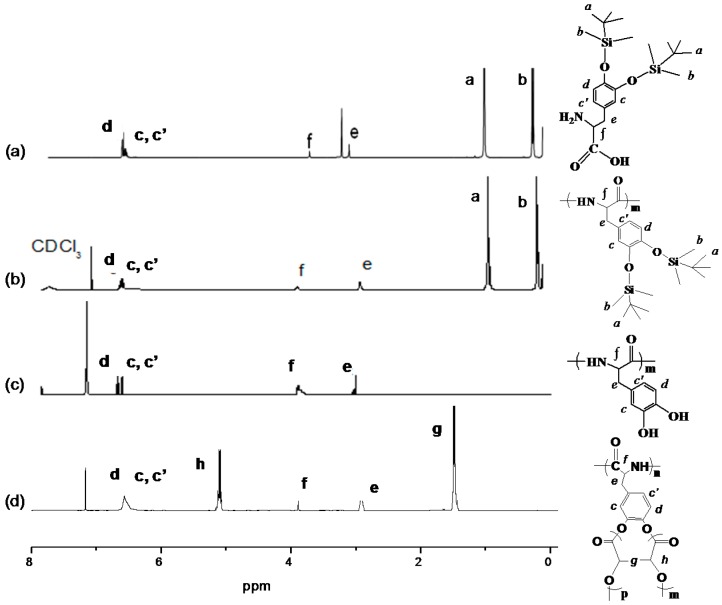
^1^H NMR spectra of DOPA(TBDMS)_2_ (**a**); PDOPA(TBDMS)_2_ (**b**); PDOPA (**c**); and PDOPA-*g*-PLA_20_ (**d**).

To avoid the oxidation of the catechol groups during polymerization, the hydroxyl groups in DOPA was firstly protected by TBDMS (Scheme 1) to obtain DOPA(TBDMS)_2_. ^1^H NMR spectrum of DOPA(TBDMS)_2_ is shown in Figure 1a. Chemical shifts at 0.2 and 1.01 ppm assigned to the methyl of the butyldimethylsilyl groups. The chemical shifts at 2.95, 3.85, and 6.6–6.8 ppm belonged to the methylene, methane, and benzene groups in DOPA, respectively. Then, PDOPA(TBDMS)_2_ oligomer could be prepared in the presence of EDC and HOBT. The ^1^H NMR spectrum confirmed the structure of the PDOPA(TBDMS)_2_ polymer (Figure 1b). By deprotection, the TBDMS groups were removed and the chemical shifts of the methyl groups were disappeared in ^1^H NMR spectrum (Figure 1c). Finally, the PDOPA-*g*-PLA (PDOPA-*g*-PLA_20_ as an example) copolymer was prepared by ROP of lactide using PDOPA as initiator. In the ^1^H NMR spectrum (Figure 1d), new peaks appeared at 1.5 and 5.1 ppm assigned to the methyl and methine groups, which indicated that the graft PDOPA-*g*-PLA copolymer with comb-like structure was successfully prepared. By changing the molar ratio of the initiator PDOPA and the monomer LA, a series of the PDOPA-*g*-PLA copolymers with various LA compositions were synthesized and listed in Table 1. GPC measurements showed that the molecular weight (*M*_n_) of the PDOPA oligomer was 0.2 × 10^4^, and *M*_n_ of the PDOPA-*g*-PLA copolymers were increased from 0.6 × 10^4^ to 1.3 × 10^4^ with increment of the LA composition. However, *M*_n_ could not further increase when the molar ratio of n_PDOPA_:n_LA_ was 1:50, possibly due to the steric hindrance of the DOPA units.

**Figure 2:**
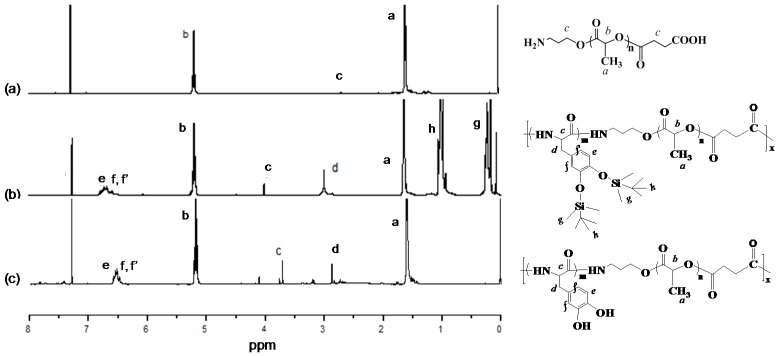
^1^H NMR spectra of H_2_N–PLA–COOH (**a**); PDOPA(TBDMS)_2_-*b*-PLA_20_ (**b**); and PDOPA-*b*-PLA_20_ (**c**).

In order to prepare the PDOPA-*b*-PLA block copolymer, H_2_N-PLA-COOH oligomer was firstly synthesized by ROP of lactide using amino propanol as initiator and followed carboxylation with succinic anhydride [37]. The PDOPA-*b*-PLA copolymer was synthesized by amidation reaction of PDOPA(TBDMS)_2_ and H_2_N-PLA-COOH, as illustrated in Scheme 2, and subsequently deprotected the hydroxyl groups by TBAF. The structures of the H_2_N-PLA-COOH oligomer and the PDOPA-*b*-PLA (PDOPA-*b*-PLA_20_ as an example) copolymers were characterized by ^1^H NMR spectra, as shown in Figure 2. The chemical shifts at 5.1 and 1.5 ppm were ascribed to the protons of the methine and methyl groups in LA units (Figure 2a). For the PDOPA(TBDMS)_2_-*b*-PLA copolymer (Figure 2b), peaks appearing at 3.8, 4.2, and 6.8 ppm assigned to the –CH_2_Ar, –HNC(H)CO–, and –Ar groups in DOPA units, respectively, and peaks at 0.2 and 1.01 ppm assigned to the methyl of the TBDMS groups, indicating the formation of the PDOPA(TBDMS)_2_-*b*-PLA copolymer. After removing the TBDMS groups, its chemical shifts at 0.2 and 1.01 ppm were disappeared (Figure 2c), suggesting the deprotection of the hydroxyl groups in DOPA. By changing the molar ratios of the PLA and PDOPA(TBDMS)_2_ oligomers ranged from 50:1 to 1:50, the various PDOPA-*b*-PLA copolymers were obtained (Table 2).

The molecular weights of the PDOPA-*b*-PLA copolymers were 1.3 × 10^4^–2.3 × 10^4^ from the GPC measurements (Figure S1), which were higher than the graft copolymers. There are only about 11 DOPA units formed in one PDOPA oligomer chain (*M*_n_ = 2000), possibly due to the steric hindrance effect between the DOPA units. According, the PDOPA-*g*-PLA copolymers had short PDOPA chains, leading the low molecular weight of the PDOPA-*g*-PLA copolymer. In the case of the PDOPA-*b*-PLA copolymers, the existing of the LA units in the backbone might decrease the steric hindrance of the PDOPA units, resulting in the higher molecular weights.

### 3.2. Solubility

As we known, most of the bio-based polymers such as PLA, PCL, and the natural polymers such as cellulose can only dissolve in few organic solvents, which were limited for further modifications or process. Amazingly, the resultant PDOPA-*g*-PLA and PDOPA-*b*-PLA copolymers showed good solubility in ethanol, acetone, chloroform, THF, DMF, DMSO, and other common organic solvents, as shown in Table S1. However, PLA oligomer could only dissolve in chloroform and DMF, even with the low molecular weight at 0.4 × 10^4^. This excellent solubility of the copolymers might contribute to the PDOPA chain, which could dissolve in almost all of the common organic solvents. This result indicated that the copolymers could be easy process and further modification.

### 3.3. Thermal Property

DSC measurements had been performed to investigate the effects of the structure and composition on the thermal property of the PDOPA-*g*-PLA and PDOPA-*b*-PLA copolymers and the results were summarized in Table 1 and Table 2. The melting temperature (*T*_m_) of the PDOPA oligomer was about 237 °C. Copolymerized with PLA, the *T*_m_ of the copolymers were slightly higher than the PLA polymer, possibly due to the rigid groups in PDOPA and miscibility between PLA and PDOPA domains.

**Scheme 3:**
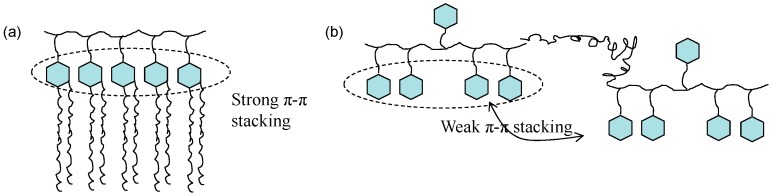
Representative schemes of the PDOPA-*g*-PLA (**a**), and PDOPA-*b*-PLA (**b**) copolymers.

**Figure 3:**
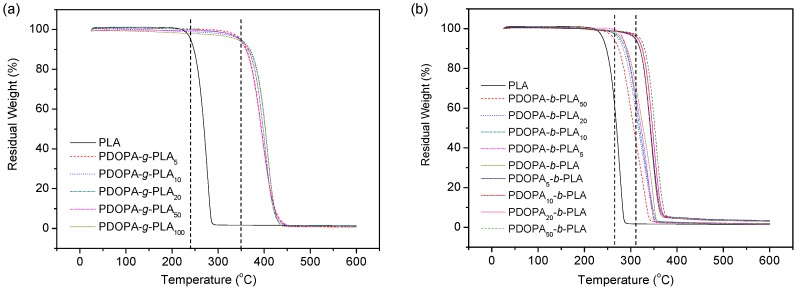
TGA curves of the PDOPA-*g*-PLA (**a**), and PDOPA-*b*-PLA (**b**) copolymers with various compositions under N_2_.

TGA curves of the PDOPA-*g*-PLA and PDOPA-*b*-PLA copolymers are shown in Figure 3. The decomposition temperature at 5% weight loss (*T*_d,5%_) that calculated from in Figure 3 are also listed in Table 1 and Table 2. Weight loss does not take place for all copolymers before 200 °C under N_2_ atmosphere. The *T*_d,5%_ of the PDOPA-*g*-PLA copolymers was 341~350 °C, which was much higher than the PLA homopolymer (*M*_n_ = 0.4 × 10^4^, 240 °C). For the PDOPA-*b*-PLA copolymers, the *T*_d,5%_ was ranged from 263 to 320 °C depending on the PDOPA compositions. With increasing the PDOPA composition in both two kinds of graft and block copolymers, the *T*_d,5%_ increased, indicating the contribution of the PDOPA chains for the enhanced thermal stability of the copolymers. By comparing the graft and block copolymers, the graft copolymers showed more excellent thermal stability. Since the backbone of the graft copolymers was composed of the rigid DOPA units, the comb-like structure of the PDOPA-*g*-PLA copolymers was relatively regular and there were strong π-π stacking interactions among the intermolecular and intramolecular chains (Scheme 3a). On the contrary, the PDOPA-*b*-PLA copolymers, special for the PLA segments showed the linear structure. Moreover, the phenol moieties might have the specific interactions such as hydrogen interactions with PLA chains, and leading the miscibility of the two segments of PLA and PDOPA [40]. Accordingly, the structure of PDOPA-*b*-PLA copolymers was more irregular. Thus, the π-π stacking interactions among the intermolecular and intramolecular chains were relatively weaker, compared to the PDOPA-*g*-PLA copolymers (Scheme 3b). As a result, the thermal stability of the PDOPA-*g*-PLA copolymers was higher than the PDOPA-*b*-PLA copolymers.

### 3.4. Hydrolytic Degradation

**Figure 4:**
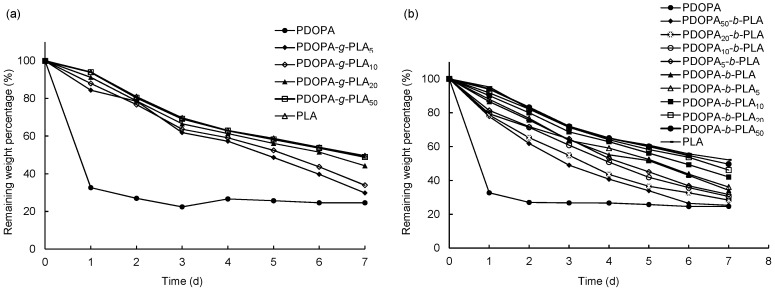
Remaining weights of PDOPA-*g*-PLA (**a**) and PDOPA-*b*-PLA (**b**) with degradation time in buffer solution at pH 10.2.

Degradation of the bio-based polymers is a very important factor for the materials to the biomedical applications. Therefore, the hydrolytic degradation properties of the PDOPA-*g*-PLA and PDOPA-*b*-PLA copolymers were investigated in alkaline buffer at pH 10.2 as an accelerated test. The buffers were used in excess to avoid auto-catalysis and maintain a stable pH environment. The degradation behavior was detected by the weight loss during the hydrolysis, as shown in Figure 4. In the first period of degradation for one day, the weight loss of the PDOPA was very quickly, about 70%. Then, the PDOPA kept almost stable even degradation for seven day. This stable weight might be induced by the formation of the oxidation and crosslinking structure of the DOPA units in alkaline buffer solution, as reported in the previous reports [27,29,36]. The remaining weights of the PDOPA-*g*-PLA and PDOPA-*b*-PLA copolymers were slowly decreased with increasing the degradation time. Comparing the PDOPA-*g*-PLA and PDOPA-*b*-PLA copolymers, the PDOPA-*g*-PLA copolymers showed quicker degradation rate, possibly due to the shorter molecular chains. Moreover, the degradation rate of the two type copolymers (especial PDOPA-*b*-PLA copolymers) was depended on the DOPA composition, and quicker with increasing the DOPA composition. Therefore, the PDOPA-*g*-PLA and PDOPA-*b*-PLA copolymers showed biodegradability and the rate could be adjusted by the DOPA composition.

**Figure 5:**
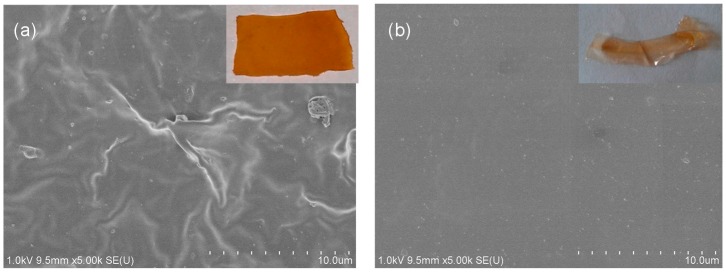
SEM and photo images of the PDOPA-*g*-PLA_20_ copolymer films before (**a**) and after (**b**) degradation.

Interestingly, the copolymer films after degradation became light yellow and transparent (inset images in Figure 5b), compared to the non-degraded copolymers with opaque and brown color (inset images in Figure 5a). Then, the surface morphologies of the copolymer films before and after degradation (PDOPA-*g*-PLA_20_ as an example) were investigated by SEM observations, as shown in Figure 5. Before degradation, the copolymer film showed many wrinkles on the surface in Figure 5a. After degradation, the film thickness became thin. However, surprisingly, the film surface changed to be less wrinkled (Figure 5b). This result was opposite to the previous results; that is, the surface of the copolymer film generally became rougher and appeared micro-/nano-pores [24]. Moreover, the results from the solubility tests showed that the PDOPA-*g*-PLA_20_ copolymer after degradation could not dissolve but swell in DMF, THF, and other common organic solvents (Table S2). In contrast, the copolymer before degradation showed good solubility in common organic solvents. In our previous report, DOPA was proved to degrade into the monomer and oligomers, the oligomer possibly being the melanine after auto-oxidation of DOPA monomers in pH 10.2 buffer solution [36]. Thus, the results of the less wrinkled surface and the low solubility might be due to the formation of the oxidation and crosslinking structure of the PDOPA during degradation in the basic condition. The mechanism of the degradation is now further proved. Since the degraded copolymer undissolved in solvents, the structure and molecular weight of the remaining copolymer after degradation was not detected.

### 3.5. Biocompatibility

**Figure 6:**
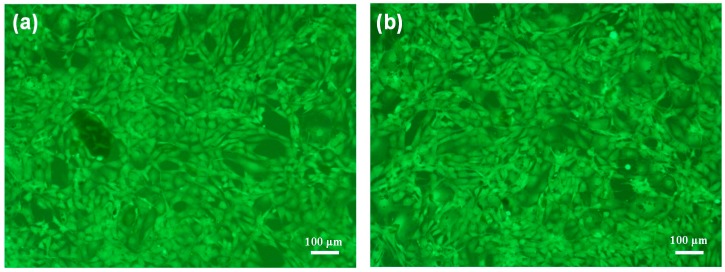
Fluorescence microscopy images of NIH/3T3 cells grown on the PDOPA-*g*-PLA_5_ (**a**) and PDOPA-*b*-PLA_5_ (**b**) copolymers.

The cytotoxicities of the PDOPA-*g*-PLA and PDOPA-*b*-PLA copolymers were estimated using NIH/3T3 cells as the model cells, and the morphology of the cell growth was also observed by fluorescence microscopy. Figure 6 shows the NIH/3T3 cell adhesion on the copolymer surfaces. It was clear that the NIH/3T3 cells adhered and proliferated on both the PDOPA-*g*-PLA and PDOPA-*b*-PLA copolymers, indicating the good cell biocompatibility of the PDOPA-*g*-PLA and PDOPA-*b*-PLA copolymers, as our previous report [37].

## 4. Conclusions

Two bio-based graft and block PDOPA-*g*-PLA and PDOPA-*b*-PLA copolymers were polymerized based on LA and DOPA. The molecular weight and composition of the copolymers could be adjusted by changing the molar ratio of LA and DOPA. All the copolymers with different structures showed high solubility even in common organic solvents. The studies on the thermal and degradable properties suggested that the polymer structures affected their properties. The PDOPA-*g*-PLA copolymers showed higher thermal stability while quicker degradation speed than the PDOPA-*b*-PLA copolymers, due to the PDOPA-*g*-PLA copolymers with regular structure and short main chain. The degraded copolymer was undissolved but swelled in the common organic solvents because of the crosslinking formation of the PDOPA chains at basic condition. Moreover, the both PDOPA-*g*-PLA and PDOPA-*b*-PLA copolymers showed good biocompatibility. Unfortunately, the copolymers showed low mechanical properties. Further research into the mechanism of the degradation and the enhancement of the mechanical properties are now being explored.

## Figures and Tables

**Table 1:** Synthesis and characterization of the PDOPA-*g*-PLA copolymer.

Sample	n_PDOPA_:n_LA_	*M*_n_ × 10^−4 a^	*M*_w_/*M*_n_^a^	*T*_m_ (°C) ^b^	*T*_d,5%_ (°C) ^c^
PDOPA	/	0.2	1.24	237	/
PDOPA-*g*-PLA_5_	1:5	0.6	1.19	169	350
PDOPA-*g*-PLA_10_	1:10	0.8	1.26	171	349
PDOPA-*g*-PLA_20_	1:20	1.0	1.41	173	349
PDOPA-*g*-PLA_50_	1:50	1.3	1.60	175	345
PDOPA-*g*-PLA_100_	1:100	1.3	1.79	175	341
PLA	/	0.4	1.18	162	240

^a^ Molecular weight results (*M*_n_ and *M*_w_/*M*_n_) were determined by GPC measurement using THF as eluent; ^b^ Results from DSC measurement; ^c^ Results from TGA measurement.

**Table 2:** Synthesis and characterization of the PDOPA-*b*-PLA copolymer.

Sample	n_PDOPA_: n_PLA_	*M*_n_× 10^−4 a^	*M*_w_/*M*_n_^a^	*T*_m_ (°C) ^b^	*T*_d,5%_ (°C) ^c^
PDOPA	/	0.2	1.24	237	/
PDOPA_50_-*b*-PLA	50:1	2.0	1.20	180	321
PDOPA_20_-*b*-PLA	20:1	2.0	1.27	180	316
PDOPA_10_-*b*-PLA	10:1	1.8	1.23	178	310
PDOPA_5_-*b*-PLA	5:1	1.5	1.29	175	309
PDOPA-*b*-PLA	1:1	1.3	1.44	171	281
PDOPA-*b*-PLA_5_	1:5	1.5	1.35	174	283
PDOPA-*b*-PLA_10_	1:10	1.8	1.33	177	277
PDOPA-*b*-PLA_20_	1:20	2.2	1.35	179	273
PDOPA-*b*-PLA_50_	1:50	2.3	1.44	181	263
PLA	/	0.4	1.18	162	240

^a^ Molecular weight results (*M*_n_ and *M*_w_/*M*_n_) were determined by GPC measurement using THF as eluent; ^b^ Results from DSC measurement; ^c^ Results from TGA measurement.

## Supplementary Materials

The following are available online at www.mdpi.com/2073-4360/8/3/92/s1. Table S1. Solubilities of the PDOPA and PLA polymers and their copolymers; Figure S1. GPC traces of the PDOPA-*b*-PLA (**a**); PLA (**b**) and PDOPA (**c**); Table S2. Properties of the PDOPA-*g*-PLA20 copolymer film before and after degradation.

## References

[1] Coombs J., Hall K. (1998). Chemicals and polymers from biomass. Renew. Energ..

[2] Mülhaupt R. (2013). Green polymer chemistry and bio-based plastics: Dreams and reality. Macromol. Chem. Phys..

[3] Fernandez J., Etxeberria A., Varga A.L., Sarasua J.R. (2015). Synthesis and characterization of ω-pentadecalactone-*co*-ε-decalactone copolymers: Evaluation of thermal, mechanical and biodegradation properties. Polymer.

[4] Babu R.P., O’Connor K., Seeram R. (2013). Current progress on bio-based polymers and their future trends. Prog. Biomater..

[5] Furkan H., Isikgor C., Remzi B. (2015). Lignocellulosic biomass: A sustainable platform for the production of bio-based chemicals and polymers. Polym. Chem..

[6] Zeng C., Seino H., Ren J., Hatanaka K., Yoshie N. (2013). Bio-based furan polymers with self-healing ability. Macromolecules.

[7] Nikolau B.J., Perera M.A., Brachova L., Shanks B. (2008). Platform biochemicals for a biorenewable chemical industry. Plant. J..

[8] Mialon L., Vanderhenst R., Pemba A.G., Miller S.A. (2011). Polyalkylenehydroxybenzoates (PAHBs): Biorenewable aromatic/aliphatic polyesters from lignin. Macromol. Rapid Commun..

[9] Dong W.F., Ren J.J., Shi D.J., Ma P.M., Li X., Duan F., Ni Z.B., Chen M.Q. (2013). Hydrolyzable and bio-based polyester/nano-hydroxyapatite nanocomposites: Sructure and properties. Polym. Degrad. Stab..

[10] Shoichet M.S. (2009). Polymer scaffolds for biomaterials applications. Macromolecules.

[11] Hartmann M.H. (1998). High molecular weight polylactic acid polymers. Biopolymers from Renewable Resources.

[12] Al-Itry R., Lamnawar K., Maazouz A. (2015). Biopolymer blends based on poly(lactic acid): Shear and elongation rheology/structure/blowing process relationships. Polymers.

[13] Blanco I. (2014). End-life prediction of commercial PLA used for food packaging through short term TGA experiments: Real chance or low reliability?. Chin. J. Polym. Sci..

[14] Hassan E., You W., He J., Yu M.H. (2013). Dynamic mechanical properties and thermal stability of poly(lactic acid) and poly(butylene succinate) blends composites. J. Fiber Bioengin. Inform..

[15] Blanco I., Siracusa V. (2013). Kinetic study of the thermal and thermo-oxidative degradations of polylactide-modified films for food packaging. J. Therm. Anal. Calorim..

[16] Ma P.M., Shen T.F., Xu P.W., Dong W.F., Lemstra P.J., Chen M.Q. (2015). Superior performance of fully biobased poly(lactide) via stereocomplexation-induced phase separation: Structure *versus* property. ACS. Sustain. Chem. Eng..

[17] Stout D.A., Basu B., Webster T.J. (2011). Poly(lactic-*co*-glycolic acid): Carbon nanofiber composites for myocardial tissue engineering applications. Acta. Biomater..

[18] Teng C.Q., Yang K., Ji P., Yu M.H. (2004). Synthesis and characterization of poly(l-lactic acid)-poly(ε-caprolactone) multiblock copolymers by melt polycondensation. J. Polym. Sci. A Polym. Chem..

[19] Chen G.X., Kim H.S., Shim J.H., Yoon J.S. (2005). Role of epoxy groups on clay surface in the improvement of morphology of poly(l-lactide)/clay composites. Macromolecules.

[20] Ma P.M., Jiang L., Xu P.W., Dong W.F., Chen M.Q., Lemstra P.J. (2015). Rapid stereocomplexation between enantiomeric comb-Shaped cellulose-*g*-poly(l-lactide) nanohybrids and poly(d-lactide) from the melt. Biomacromolecules.

[21] Li F., Li S.M., Ghzaoui A.E., Nouailhas H., Zhuo R.X. (2007). Synthesis and gelation properties of PEG-PLA-PEG triblock copolymers obtained by coupling monohydroxylated PEG-PLA with adipoyl chloride. Langmuir.

[22] Wang J., Zheng L.C., Li C.C., Zhu W.X., Zhang D., Guan G.H., Xiao Y.N. (2014). Synthesis and properties of biodegradable multiblock poly(ester-carbonate) comprising of poly(l-lactic acid) and poly(butylene carbonate) with hexamethylene diisocyanate as chain-extender. J. Appl. Polym. Sci..

[23] Xu Y.P., Li J.H., Chen M.Q., Ren J.J., Ni Z.B., Liu X.Y. (2010). Synthesis and characterization of biodegradable polyester P(DHCA-*co*-LA). Acta. Polym. Sin..

[24] Shi D.J., Hua J.T., Zhang L., Chen M.Q. (2015). Synthesis of bio-based poly(lactic acid-*co*-hydroxy decanonate) copolymers with thermal stability and ductility. Polymers.

[25] Sedó J., Saiz-Poseu J., Busqué F., Ruiz-Molina D. (2013). Catechol-based biomimetic functional materials. Adv. Mater..

[26] Moulay S. (2014). Dopa/catechol-tethered polymers: Bioadhesives and biomimetic adhesive materials. Polym. Rev..

[27] Lee H., Dellatore S.M., Miller W.M., Messersmith P.B. (2007). Mussel-inspired surface chemistry for multifunctional coatings. Science.

[28] Yu J., Wei W., Menyo M.S., Masic A., Waite J.H., Israelachvili J.N. (2013). Adhesion of mussel foot protein-3 to TiO_2_ surfaces: The effect of pH. Biomacromolecules.

[29] Postma A., Yan Y., Wang Y., Zelikin A.N., Tjipto E., Caruso F. (2009). Self-polymerization of dopamine as a versatile and robust technique to prepare polymer capsules. Chem. Mater..

[30] Mrówczyński R., Nan A., Turcu R., Leistner J., Liebscher J. (2015). Polydopamine–A versatile coating for surface-initiated ring-opening polymerization of lactide to polylactide. Macromol. Chem. Phys..

[31] Ahn B.K., Das S., Linstadt R., Kaufman Y., Martinez-Rodriguez N.R., Mirshafian R., Kesselman E., Talmon Y., Lipshutz B.H., Israelachvili J.N. (2015). High-performance mussel-inspired adhesives of reduced complexity. Nature Commun..

[32] Ahn B.K., Lee D.W., Israelachvili J.N., Waite J.H. (2014). Surface-initiated self-healing of polymers in aqueous media. Nat. Mater..

[33] Shi D.J., Liu R.J., Dong W.F., Li X.J., Zhang H.J., Chen M.Q., Akashi M. (2015). pH-Dependent and self-healing properties of mussel modified PVC hydrogels in metal-free environment. RSC Adv..

[34] Chen X., Yan Y., Müllner M., Koeverden M.P., Noi K.F., Zhu W., Caruso F. (2014). Engineering fluorescent poly(dopamine) capsules. Langmuir.

[35] Lee H., Lee B.P., Messersmith P.B. (2007). A reversible wet/dry adhesive inspired by mussels and geckos. Nature.

[36] Shi D.J., Liu R.J., Ma F.D., Chen D.Y., Chen M.Q., Akashi M. (2014). Studies on synthesis, characterization and functionalization of poly(3,4-dihydroxy-l-phenylalanine). Chem. Lett..

[37] Shi D.J., Zhang L., Shen J.L., Li X.J., Chen M.Q., Akashi M. (2015). Fabrication of rod-like nanocapsules based on polylactide and 3,4-dihydroxyphenylalanine for drug delivery system. RSC Adv..

[38] Sever M.J., Wilker J.J. (2001). Synthesis of peptides containing DOPA (3,4-dihydroxyphenylalanine). Tetrahedron.

[39] Deng C., Chen X., Yu H., Sun J., Lu T., Jing X. (2007). A biodegradable triblock copolymer poly(ethylene glycol)-*b*-poly(lactide)-*b*-poly(l-lysine): Synthesis, self-assembly, and RGD peptide modification. Polymer.

[40] Meaurio E., Zuza E., Sarasua J.R. (2005). Miscibility and specific interactions in blends of poly(l-lactide) with poly(vinylphenol). Macromolecules.

