# Supplementary material for: Studies on Preparation of Poly(3,4-Dihydroxyphenylalanine)-Polylactide Copolymers and the Effect of the Structure of the Copolymers on Their Properties
Source: Polymers (Basel). 2016 Mar 18;8(3):92. doi: 10.3390/polym8030092 (PMC6432557; doi:10.3390/polym8030092)
Supplement: Supplementary file 1 [file polymers-08-00092-s001.pdf]

Supplementary Materials: Studies on Preparation of Poly(3,4-Dihydroxyphenylalanine)-Polylactide Copolymers and Effect of the Structure of the Copolymers on Their Properties

Dongjian Shi, Jiali Shen, Zenghui Zhao, Chang Shi and Mingqing Chen

The PLA-PDOPA copolymer showed well solubility in ethanol, acetone, chloroform, THF, DMF, DMSO and other common organic solvents, as shown in Table S1. While PLA oligomer could only dissolve in chloroform and DMF. This excellent solubility of the PLA-PDOPA copolymers might contribute to the PDOPA chain, which could dissolve in almost all of the common organic solvents.

Table S1. Solubilities of the PDOPA and PLA polymers and their copolymers.

Solvent	PDOPA	PDOPA-*g*-PLA$_5$	PDOPA-*b*-PLA$_5$	PLA
Water	×	×	×	×
Chloroform	○	○	○	○
Ethanol	○	○	○	×
Acetone	○	○	○	△
Acetyl ether	○	○	○	×
THF	○	○	○	△
DMSO	○	○	○	△
DMF	○	○	○	○

○: dissolve, △: part dissolve, ×: not dissolve

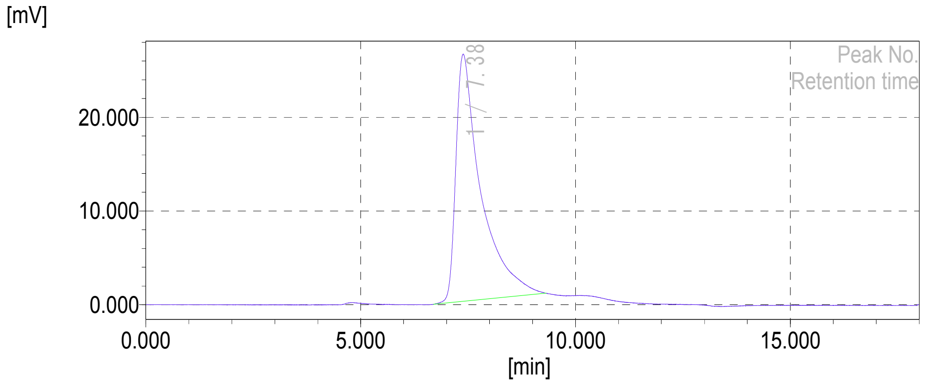

(**a**)

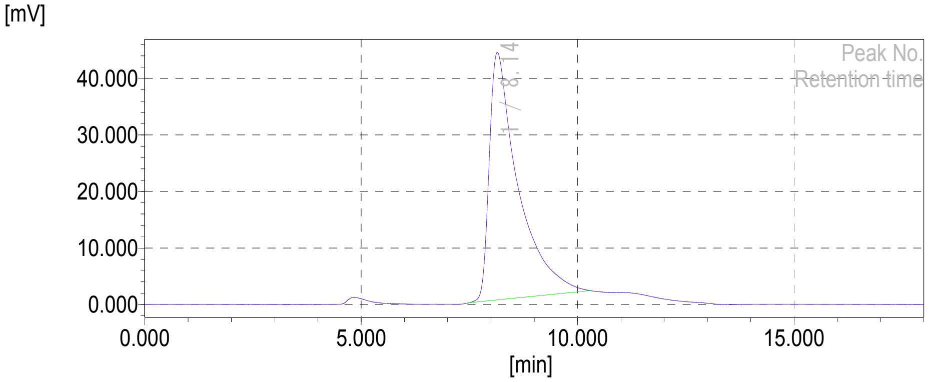

(**b**)

Figure S1. *Cont.*

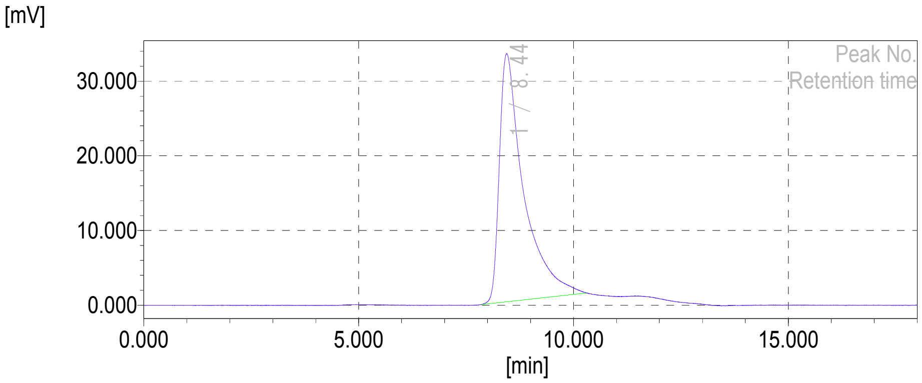

(**c**)

Figure S1. GPC traces of the PDOPA-*b*-PLA (**a**); PLA (**b**) and PDOPA (**c**).

Table S2. Properties of the PDOPA-*g*-PLA_{20} copolymer film before and after degradation.

PDOPA-*g*-PLA_{20}	Color	Solubility	M_n×10^{-4} [a]
Before	Yellow-brown, opaque	Dissolve in DMF, THF, ...	1.0
After	Yellow, transparent	Swell in DMF, THF,...	1.7

[a] From GPC measurement using DMF as eluent and mono-dispersed polystyrene as standards. Since the copolymer after degradation was swollen in DMF, M_n of the copolymer after degradation was measured the supernatant solution of the copolymer in DMF.

© 2016 by the authors; licensee MDPI, Basel, Switzerland. This article is an open access article distributed under the terms and conditions of the Creative Commons by Attribution (CC-BY) license (http://creativecommons.org/licenses/by/4.0/).
